# Mind full of kindness: self-awareness, self-regulation, and self-transcendence as vehicles for compassion

**DOI:** 10.1186/s40359-022-00888-4

**Published:** 2022-07-29

**Authors:** Jacob T. Miller, Paul Verhaeghen

**Affiliations:** School of Psychology, Georgia Institute of Technology, 654 Cherry Street NW, Atlanta, GA 30332 USA

**Keywords:** Mindfulness, Compassion, Moral foundations, Awareness of privilege

## Abstract

**Background:**

We investigated the relationship between mindfulness and compassion in a broader way than is typically done by (a) using a recent, comprehensive conceptualization of mindfulness as a manifold of self-awareness, self-regulation, and self-transcendence, and (b) by casting a wide net of compassion measures [i.e., the Compassionate Love for Humanity Scale (Sprecher and Fehr in J Soc Pers Relatsh 22(5):629–651, 2005); Compassion Scale (Martins et al. in J Health Care Poor Underserved 24:1235–1246, 2013); Compassion Scale (Pommier in Assessment 27:21–39, 2020); Relational Compassion Scale (Hacker in The relational compassion scale: Development and validation of a new self-rated scale for the assessment of self-other compassion, University of Glasgow, 2008); and the SOCS-O scale (Gu et al. in Clin Psychol Rev 37:1–12, 2020)]. Additionally, we examined the interplay between mindfulness, compassion, and ethical sensitivities by assessing the influence of the moral foundations (individualizing and binding) on compassion, and the influence of mindfulness, the moral foundations, and compassion on awareness of privilege.

**Methods:**

We surveyed 407 undergraduate students. Factor analysis was used to examine the dimensionality of the compassion measures; path analysis to examine the relationships between all variables.

**Results:**

Factor analysis revealed distinct affective (empathy, indifference), cognitive (common humanity, recognizing suffering), and motivational (willingness to act) aspects of compassion. Mindfulness, under its aspects of reflective awareness, self-compassion, and self-transcendence, was associated with compassion, with reflective awareness predicting multiple aspects of compassion over and beyond the normal mechanisms of the mindfulness manifold and the moral foundations. Individualizing was associated with all aspects of compassion; binding was only connected to recognizing suffering and a willingness to act. Awareness of privilege was positively connected to mindfulness through individualizing and the recognition of common humanity; it was also directly negatively related to the moral foundation of binding.

**Conclusions:**

Mindfulness and compassion have synergistic and distinct positive effects on ethical sensitivities. Given that both compassion and ethical sensitivities have roots in mindfulness, mindfulness interventions might be one possible venue to enhance these positive aspects of individuals’ psychology.

## Background

The ability to feel compassion–essentially, being touched by the suffering of others, coupled with the wish to relieve it [1]–is an important asset in a social species such as ours. Here, we investigate a natural but under-studied antecedent of compassion, namely trait mindfulness, that is, the ability or propensity to engage in “nonelaborative, non-judgmental, present-centered awareness in which each thought, feeling, or sensation that arises in the attentional field is acknowledged” [2, p. 232]. As pointed out by Khoury [3], many of the current definitions of compassion include elements of what is traditionally considered mindfulness, such as noticing or awareness of distress or suffering [1] and a non-judgmental stance towards strangers [4]. Our study aims to investigate this connection in a wider perspective than is typically done, that is, both by using a recent, broad conceptualization of mindfulness as a manifold of self-awareness, self-regulation, and self-transcendence, and by casting a wide net of compassion measures, including its affective, cognitive, and conative components. This, then, allows for a more detailed look at possible psychological mechanisms to explain these triple facets of compassion. Our interest goes beyond mere academic curiosity. Compassion is a critical prosocial attitude and/or behavior, and knowledge of its antecedents, especially if they are as trainable as mindfulness is [5], might be of vital importance to society.

### The mindfulness manifold

Recent theoretical work within the field of mindfulness [6] has concluded that mindfulness is a multifaceted concept–a manifold (or perhaps even a cascade of processes) of distinct yet interrelated constructs rather than a singular concept. This work has capitalized on the finding that mindfulness is related to a multitude of positive outcomes: Mindfulness interventions lead to lower levels of stress, higher levels of well-being, decreased anxiety, depression, and negative emotions, more effective emotion regulation, less rumination, heightened self-compassion, and increased empathy [7]. There is good evidence that mindfulness may be the causal factor here: Individuals who show larger gains in dispositional mindfulness after meditation training also show decreased self-perceived stress, depressed mood, anxiety, negative affect, rumination, and increased positive affect and general well-being [8, 9].

The translation of nonelaborative, non-judgmental, present-centered awareness into positive outcomes has received much attention in the literature. Generally, three categories of potential mechanisms have been proposed, as Vago and Silbersweig [6] point out. A first such mechanism is what is typically or traditionally meant by mindfulness: a change in *self-awareness*, that is, the recognition of ingrained habits and patterns of reactivity, accompanied by enhanced awareness of momentary states of body and mind. Changes in *self-regulation,* that is, improved emotion regulation, enhanced self-compassion, and greater nonattachment and acceptance, form a second proposed mechanism. Changes in *self-transcendence* are the final mechanism, and this includes increased decentering as well as greater cognizance of the interdependence between self and others. This common-denominator model has been labeled the S-ART model, after its three components: self-awareness, self-regulation, and self-transcendence [6].

Our empirical work on the subject [10–13], based on exploratory and confirmatory factor analysis of a wide variety of measures, confirmed the plausibility of the S-ART mindfulness manifold and suggested a set of cascading processes, where self-awareness influences self-regulation which in turn influences self-transcendence. Factor analysis revealed additional subcomponents within the mechanisms of self-awareness and self-regulation. That is, self-awareness was found to consist of both an active, deliberate, probing aspect (which we labeled reflective awareness) and a more passive, equanimous, non-judgmental aspect of mindfulness (which we labeled controlled sense-of-self in the moment. Self-regulation was revealed to include (the opposite of) self-preoccupation as well as self-compassion. (More details on how to measure these aspects can be found in the Methods section.)

### Compassion

As is the case with mindfulness, many current conceptualizations of compassion (e.g., [3, 14, 15] view compassion as a conglomerate of different components, which can be usefully subsumed under affective, cognitive/perceptual, and motivational/behavioral factors. Probably the most detailed narrative review of the compassion literature (Strauss et al.) concludes that compassion can be captured in five elements: (a) recognizing suffering,(b) understanding the universality of human suffering, or understanding our common humanity; (c) feeling for, or empathy with the person suffering; (d) tolerating uncomfortable feelings; and (e) motivation to act. The former two are cognitive components, the two following components are affective, and the latter is motivational. Straus et al. note that traditional assessment instruments often concentrate on only a few of these elements. Their research group has therefore built a comprehensive survey instrument that contains all five of their proposed factors–the Sussex-Oxford Compassion Scales–consisting of a subscale for self-compassion and one for other-oriented compassion (SOCS-S and SOCS-O; [16]).

### Links between mindfulness and compassion

The empirical literature on the links between mindfulness and compassion is sparse. The one extant meta-analysis on the correlations between trait mindfulness and prosocial behavior [17] identified 12 such studies, of which only two included measures of compassion (viz., [18, 19]). The median correlation between mindfulness and compassion in these studies was 0.36. The latter study found a link between mindfulness and empathy, kindness, and common humanity, suggesting that at least the affective and cognitive aspects of compassion are affected by self-awareness. More indirect evidence for a connection comes from a meta-analysis showing that compassion-based interventions increase both compassion and mindfulness to the same extent, with effects sizes of *r* = 0.27 and 0.26, respectively [20],note that the number of studies is small, *k* = 4 and 6, resp.). Conversely, a meta-analysis on meditation interventions (which typically emphasize mindfulness) showed effects on both compassion and empathy, with effect sizes of *r* = 0.37 and 0.44, respectively ([21]; again with a small number of studies; *k* = 10 and 2, resp). Little is known on causal mechanisms, but three studies suggest a link form self-awareness to compassionate affect, and then on to real-world helping [22], caring behaviors [23] or altruistic behavior [24].

Donald et al. [17] enumerate six potential mechanisms through which a correlation between mindfulness and prosocial behavior might emerge: (a) mindfulness is associated with an increased capacity to sustain and aim attention, which might increase the likelihood of perceiving the needs of others; (b) mindfulness is often related to increased awareness of one’s bodily and emotional states, and the substrate for this awareness—the insula—is also associated with the processing of others’ emotional experiences; (c) mindfulness leads to a more positive affective experience, which in turn might be associated with greater self-reported helping behavior; (d) mindfulness enhances affect regulation, which in turn makes it less likely that compassionate responses will be inhibited and more likely to increase interpersonal warmth and kindness; (e) mindfulness helps one to perceive thoughts as mental events rather than truth, so that judgments, assumptions, and biases are less likely to guide behavior; and (f) mindfulness may alter the sense of self to a more interdependent, flexible, non-attached concept, again opening up the individual to a more prosocial attitude.

Some of those mechanisms fall naturally into the S-ART classification: Alternative (b) fits with the controlled sense-of-self aspect of self-awareness; alternative (d) would imply an involvement of the self-regulation facet; alternative (f) would imply an involvement of self-transcendence. An alternative or additional mechanism might be one we uncovered recently [13], namely that mindfulness is connected to ethical sensitivities. In that study, we investigated the relationship between mindfulness and Graham et al.’s [25] Moral Foundations Theory. This theory posits that five dimensions describe the broad concept of ethical sensitivities well: (a) promotion of care/avoidance of harm, (b) fairness; (c) ingroup loyalty; (d) respect for authority, and (e) sanctity/purity. Note that the former two dimensions focus on individual rights and are often combined into an “individualizing” foundation; the remaining three focus on processes related to ingroup cohesion and are therefore often combined into a “binding” foundation. Our data showed that the individualizing aspects of morality could be well predicted from reflective awareness and self-transcendence, but the binding aspects of morality were only directly predicted by self-transcendence and social conservatism. Individuals who took a more individualizing stance, in turn, exhibited more awareness of privilege, less explicit prejudice, and displayed a higher motivation to control their prejudiced reactions. The binding stance, in contrast, led to more prejudice and less awareness of privilege, but also a higher motivation to control one’s prejudiced reactions (possibly through a different mechanism, that is, the need to dissimulate). A possible prediction then could be that individualizing would positively relate to compassionate attitudes, and binding would possibly negatively relate to such attitudes, at least when compassion is operationalized—as is done here—as compassion towards strangers.

As explained above, In the Verhaeghen and Aikman [13] study, we found that mindfulness and the moral foundations were related to different outcomes related to bias and prejudice. Here we picked one of those outcomes—awareness of privilege—in order to examine if compassion mediates this relationship. Awareness of privilege was chosen because it was the only variable whose influence from mindfulness was fully mediated through the moral foundations. Given our suspicion that the moral foundations are related to compassion, we were eager to examine whether compassion mediated between the moral foundations and awareness of privilege.

### Measures of compassion

Given that our study is one of the first of its kind, we cast a wide net of compassion measures in order to capture the construct in as many of its facets as possible. To assess the structure of compassion inherent in these measures, we conducted a factor analysis at the item level. Strauss et al.’s [15] and Khoury’s [3] meta-analyses on compassion were used as sources to identify potential scales. Both meta-analyses mention the Compassionate Love for Humanity Scale (CLH, [26], Santa Clara Brief Compassion Scale (SCBCS, [27], Compassion Scale by Martins et al. (CS-M, [28], Compassion Scale by Pommier (CS-P; [29], Self-Compassion Scale (SCS, [30], Relational Compassion Scale (RCS, [31], Compassionate Care Assessment Tool (CCAT, [32], and Schwartz Center Compassionate Care Scale (SCCCS, [33]. Additionally, we included the more recent SOCS-O scale [16]. The scales we retained are the CLH, CS-M, CS-P, RCS, and SOCS-O. Scales not included in our study are the CLS-CO, SCBCS, SCS, CCAT, SCCCS, and SOCS-S. CCAT and SCCCS were excluded because they were designed for use by specific populations related to medical practice. The SCBCS was excluded because it is simply a shortened version of the CLH. Finally, two scales (SCS and SOCS-S were excluded because they measure compassion toward the self rather than others,for the RCS, only the self-to-other compassion scale was used, for the same reason.

### The aim of the study

This study is mainly exploratory, in the sense that it is the first to examine the relationship between this broader concept of mindfulness with a wide range of compassion measures. Still, a number of expectations can be formulated from the literature. First, the mindfulness manifold, as in our previous papers, should yield the expected S-ART structure and its relationship with ethical attitudes, with a flow from self-awareness over self-regulation to self-transcendence, and then on the basic moral foundations of individualizing and binding. Second, the compassion surveys should yield a structure with at least three aspects—affective, cognitive, and motivational. Finally, we expect that mindfulness and compassion would be connected. From the extant literature, we expect that self-awareness will be related to at least the affective and cognitive aspects of compassion. Some of the mechanisms proposed for the mindfulness-compassion link include self-regulation and self-transcendence as well, so we might expect such connections to emerge. We also expect that part of the influence of mindfulness on compassion will be transmitted through the moral foundations as basic intuitive stances on ethical matters. Finally, we expect that compassion would be a predictor for awareness of privilege.

## Methods

### Participants

The sample consisted of 407 students at the Georgia Institute of Technology, recruited from introductory psychology classes and other psychology classes offering extra credit. This sample was a subset of a larger original sample of 479 participants. Because we planned to use exploratory factor analysis at the item level for data reduction in the compassion measures, 72 participants were removed due to missing data to avoid imputation of missing scores. Participants were ages 18–26 (mean = 19.6, SD = 1.4); 52% identified as female, the rest as male. They were compensated with course credit. The study was approved by the Institutional Review Board of the Georgia Institute of Technology.

### Measures and procedure

All surveys were presented online; participants generally took about 60 min to complete all measures. We present the measures here in a thematical grouping. Note that the structure within the mindfulness manifold (self-awareness, self-regulation, and self-transcendence) was derived after a set of exploratory and confirmatory factor analyses as reported in Verhaeghen [10].

#### Self-awareness

Self-awareness consists of two constructs. *Reflective awareness* was measured as the unit-weighted *z*-score composite of three questionnaires: (a) the Observing subscale of the Five Facets Mindfulness Questionnaire (FFMQ; [34]; 8 items); (b) the Reflectiveness subscale of the Broad Rumination Scale (BRS; Trani et al. [35]; 4 items); and (c) Search for Insight/Wisdom of the Aspects of Spirituality scale (ASP; [36], 7 items). *Controlled sense-of-self in the moment* was measured as the unit-weighted *z*-score composite of three questionnaires: (a) the Acting with Awareness subscale from the FFMQ (8 items); (b) the Sense-of-self Scale (SOSS; [37],12 items); and (c) the Nonjudging of inner experience subscale of the FFMQ (8 items).

#### Self-regulation

The first subconstruct measured within self-regulation, *self-preoccupation*, is the unit-weighted *z*-score composite of two subscales from the BRS, namely Compulsivity (5 items) and Worrying (3 items), and the Isolation (2 items) and Over-Identified (2 items) subscales of the Self-Compassion Scale, Short Form (SCS; [30]. The second subconstruct, *self-compassion,* was measured as the unit-weighted *z*-scores composite of the Self-Kindness (2 items), Common humanity (2 items), and Mindfulness (2 items) subscales of the SCS, as well as the Decentering subscale of the Experiences Questionnaire (EQ; [38],13 items).

#### Self-transcendence

Self-transcendence was measured as the unit-weighted z-score composite of the Joy (6 items) and Love (6 items) subscales of the Dispositional Positive Emotion Scale (DPES; [39], and the Meaningfulness (7 items) subscale from the Resilience Scale (RS, [40].

#### Compassion

Five scales, totaling 72 items, purporting to measure compassion were included: the Compassionate Love for Humanity Scale (CLH; [26], the Compassion Scale (CS-M, [28], the Compassion Scale (CS-P, [41], the self-to-other subscale of the Relational Compassion Scale (RCS, [31], and the Sussex-Oxford Compassion for Others Scale (SOCS-O, [16]. Participants also completed the Toronto Empathy Questionnaire (TEQ, [42], which was not included in the analyses because it appeared redundant with the empathy factor that emerged from the factor analysis of compassion scales.

Data reduction (principal component analysis with oblimin rotation) was applied to the set of compassion measures in order to facilitate both data analysis and interpretation in the structural equation path-modeling phase. The scree plot and factor interpretability suggested a 5-factor solution, which is presented in Table 3 in the “Appendix”; it explained 50% of the variance. The first factor consisted exclusively of items from the CLH. The original authors called this construct “compassionate love for humanity”, but to us it seemed to closely capture the concept of empathy. The second factor was a mixture of items from different scales, with a preponderance of items from the CP-S Indifference subscale; hence, we labeled it indifference. The third factor consisted mostly of items for the SOCS-O Understanding Universality and CS-P Common Humanity scales; we labeled it common humanity. The fourth factor contained only items from the SOC-O Recognizing Suffering subscale; accordingly, we labeled it recognizing suffering. The fifth factor consists solely of items from the CS-M; we labeled it willingness to act. Based on the results from this factor analysis, five corresponding scales were constructed for use in structural equation path modeling by subjecting the scores of each item to *z* transformation and averaging the *z*-scores for each item within a construct to yield the final score for that construct. Only items with a factor loading equal to or larger than 0.50 were used. The five compassion scales were intercorrelated, with a median absolute *r* of 0.36 (see Table 1).

**Table 1:** Correlation matrix for all relevant variables

Variable	1	2	3	4	5	6	7	8	9	10	11	12	13	14	15	16	17	18
1 Gender	1																	
2 IPIP extraversion	0.10	1																
3 IPIP agreeableness	0.17*	0.24	1															
4 IPIP conscientiousness	0.01	0.03**	0.17**	1														
5 IPIP neuroticism	0.34**	− 0.01	0.07	− 0.15**	1													
6 IPIP intellect/imagination	0.02	0.14**	0.24**	0.01	0.05	1												
7 S-A Reflective awareness	0.16**	0.12*	0.31**	0.13*	0.09	0.37**	1											
8 S-A Controlled sense-of-self in the moment	− 0.10*	0.09	0.13*	0.35**	− 0.47**	− 0.05	− 0.11*	1										
9 S-R self-compassion	− 0.10*	0.14*	0.14**	0.23**	− 0.45**	0.08	0.33**	0.26**	1									
10 S-R self-preoccupation	0.22**	− 0.11*	0.05	− 0.13*	0.61**	0.01	0.20**	− 0.56**	− 0.38**	1								
11 S-T self-transcendence	0.01	0.35**	0.38**	0.27**	− 0.34**	0.14**	0.34**	0.34**	0.54**	− 0.28**	1							
12 MFQ individualizing	0.29**	− 0.02	0.28**	0.12*	0.08	0.07	0.24**	0.01	0.13**	0.14**	0.27**	1						
13 MFQ binding	− 0.06	0.05	− 0.03	0.17**	− 0.08	− 0.14**	0.10	0.06	0.23**	− 0.02	0.23**	0.19**	1					
14 Awareness of privilege	0.39**	0.13*	0.23**	0.02	0.20**	0.07	0.19**	− 0.11*	− 0.06	0.21**	0.06	0.30**	− 0.35**	1				
15 COMP empathy	0.20**	0.12*	0.52**	0.04	0.09	0.11*	0.42**	− 0.04	0.25**	0.04	0.40**	0.38**	0.13**	0.20**	1			
16 COMP indifference	− 0.17**	− 0.19*	− 0.70**	− 0.13**	− 0.02	− 0.20**	− 0.27**	− 0.15*	− 0.10	0.01	− 0.24**	− 0.31**	0.09	− 0.25**	− 0.39**	1		
17 COMP common humanity	0.09	0.03	0.42**	0.24**	− 0.15**	0.20**	0.34**	0.08	0.36**	− 0.08	0.34**	0.40**	0.15**	0.23**	0.32**	− 0.38**	1	
18 COMP recognize suffering	0.17**	0.23**	0.41**	0.15**	0.05	0.20**	0.39**	0.04	0.24**	0.04	0.32**	0.33**	0.21**	0.18**	0.44**	− 0.35**	0.40**	1
19 COMP willingness to act	0.14**	0.07	0.17**	− 0.06	0.07	0.08	0.13*	− 0.07	0.09	0.06	0.15**	0.26**	0.21**	0.08	0.47**	− 0.16**	0.12*	0.28**

*n* = 407; IPIP = IPIP = International Personality Item Pool (ipip.ori.org); S-A = self-awareness; S-R = self-regulation; S-T = self-transcendence; MFQ = Moral Foundations Questionnaire; COMP = compassion. * *p* < .05; ** *p* < .01

#### Moral foundations

The Moral Foundations Questionnaire [25] consists of five subscales, each containing six items: (a) Harm,(b) Fairness; (c) Ingroup loyalty; (d) Authority; and (e) Purity. To simplify analyses, we collapsed the two individualizing foundations into a single construct, using unit-weighted *z*-score composites (the correlation between the two individualizing foundations was 0.52), and did the same with the three binding foundations (intercorrelations between the three binding foundations ranged from 0.46 to 0.56).

*Awareness of Privilege* was measured through the Privilege and Oppression Inventory (POI; [43], which consists of four subscales, assessing White privilege (13 items), heterosexism (10 items), Christian privilege (8 items), and sexism (8 items).

#### Control variables

Two control variables were included: personality and gender. The Mini-IPIP (IPIP; [5] measures the Big Five personality factors: Extraversion with four items for each factor.

The order in which the scales were presented was as follows: FFMQ, RS, SCS, EQ, SOSS, IPIP, BRS, DPES, ASP, CLH, CS-M, CS-P, RCS, SOCS-O, MFQ, TEQ, POI.

## Results

### Correlations

The correlation matrix is presented in Table 1. To summarize the link between mindfulness and compassion, we computed the median absolute correlations (absolute correlations were used because self-preoccupation is a reverse form of mindfulness and indifference a reverse form of compassion) between our variables of interest (precursors and outcome) and the five aspects of compassion: Reflective self-awareness had a median absolute correlation of 0.34 with compassion (all five correlations significant); for controlled sense-of-self in the moment, the median absolute correlation with compassion was 0.07 (one correlation significant); for self-preoccupation, median absolute *r* was 0.04 (none significant); for self-compassion 0.24 (three correlations significant); for self-transcendence 0.32 (all five correlations significant); for individualizing 0.33 (all five correlations significant); for binding 0.15 (four correlations significant); and finally for awareness of privilege 0.20 (four correlations significant). The aspects of compassion most strongly correlated with mindfulness were common-humanity, empathy, and recognizing suffering (median *r* = 0.34, 0.25, and 0.24, resp.); median *r* for indifference was 0.15; median *r* was 0.08 for willingness to act.

### Structural equation path modeling

For structural equation modeling (implemented in *lavaan* in R; [44] variables were organized into seven tiers to examine the flow of influence as described in the introduction. The first tier consisted of the control variables, that is, the Big Five and gender. Putting these two variables in the first tier allows us to control for the effects of personality and gender as potential underlying third variables in all other relationships. The second tier consisted of the self-awareness variables (reflective awareness and controlled sense-of-self in the moment),the second of the self-regulation variables (self-preoccupation and self-compassion); the fourth of the self-transcendence variable. The fifth tier contained the moral foundations. The sixth contained the five compassion variables. The seventh and final tier consisted of the awareness of privilege scale.

We first implemented a baseline model that included the expected flow of influence from any lower tier to all higher tiers, the assumption being that the control variables influence all other variables; self-awareness influences self-regulation, self-transcendence, compassion, the moral foundations, and awareness of privilege; and so on. Thus, all tier 1 variables connected to all variables in tiers 2–7, all tier 2 variables connect to all variables in tiers 3–7, and so on. This same analysis logic and this same model (for tiers 1–4) was used in Verhaeghen [10] and Verhaeghen and Aikman [13]. Table 1 presents the intercorrelations of the constructs used as input for the structural equation model.

**Fig. 1:**
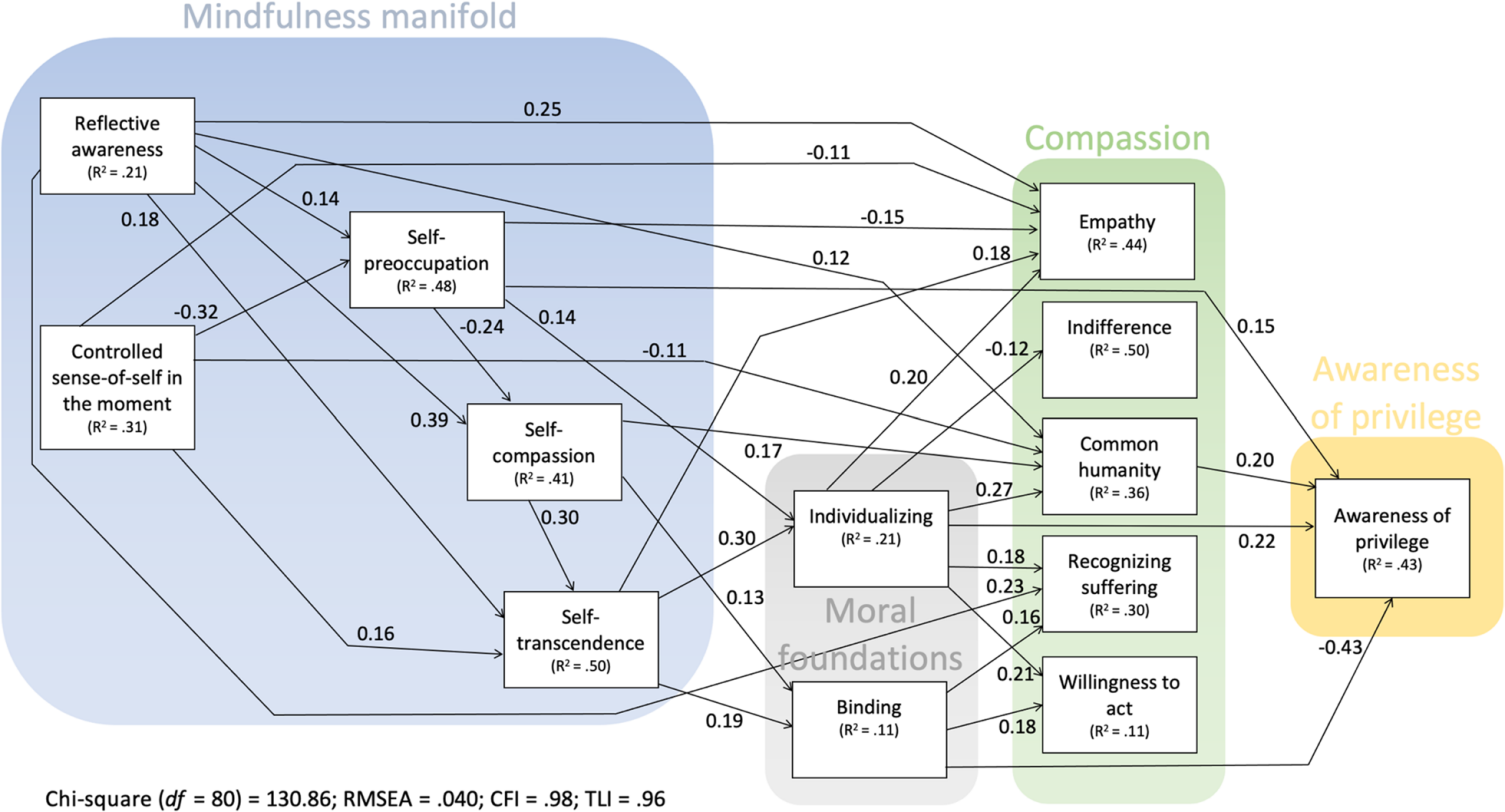
Results from path analysis, describing the relationship between the variables of interest

When implemented in *lavaan*, this model yielded poor fit: chi-square (*df* = 13) = 157.12; comparative fit index (CFI) = 0.94; Tucker-Lewis index (TLI; also known as the non-normed fit index) = 0.26; RMSEA = 0.165; SRMR = 0.033. In a second step, all non-significant paths were removed; in a third step, two paths that turned *ns* in the second step was further removed. This pruned model had excellent fit: chi-square (*df* = 80) = 149.10; CFI = 0.97; TLI = 0.94; RMSEA = 0.046; SRMR = 0.037. Modification indices suggested freeing the path from self-preoccupation to self-compassion (as in [10, 13]. This final model, depicted in Fig. 1, likewise provided excellent fit to the data: chi-square (*df* = 81) = 152.72; CFI = 0.97; TLI = 0.94; RMSEA = 0.047; SRMR = 0.037. To keep Fig. 1 relatively uncluttered, the background variables and influences therefrom were omitted from the figure; the coefficients associated with the background variables are presented in Table 2.

**Table 2:** Standardized paths from antecedent variables (the Big-Five personality factors and gender) to the mindfulness manifold, moral foundations, the compassion variables, and awareness of privilege, as estimated in the final linear structural equation model

	Extraversion	Agreeableness	Conscientiousness	Neuroticism	Intellect/ imagination	Gender
Reflective awareness		0.20	0.09		0.32	0.11
Controlled sense-of-self in the moment		0.11	0.26	− 0.43		
Self-preoccupation	− 0.10			0.45		
Self-compassion	0.06		0.10	− 0.32		
Self-transcendence	0.22	0.22		− 0.16		
Individualizing	− 0.17	0.16				0.24
Binding		− 0.10	0.11		− 0.15	
Empathy		0.37	− 0.10	0.10	− 0.14	
Indifference		− 0.66				
Common humanity		0.28	0.11	− 0.16		
Recognizing suffering	0.13	0.26				
Willingness to act		0.14	− 0.15			
Awareness of privilege	0.15				− 0.08	0.23

*N* = 407. All paths indicated are significant at *p* < .05; all paths not indicated were fixed at zero, Gender: 0 = male; 1 = female

## Discussion

The main research questions of this study were whether dispositional mindfulness, broadly construed as a manifold of self-awareness, self-regulation, and self-transcendence [6, 10], would be related to aspects of compassion, and whether the moral foundations would play a mediatory role. Additionally, we were interested to know whether compassion mediates some of the expected effects of mindfulness on awareness of privilege.

### The mindfulness manifold replicated

Before starting the discussion proper, it is important to point out that the analyses were set up to replicate the flow of influence observed in our previous studies [10, 13], which contains two studies). In those studies, the data fit the S-ART model very well, with a few sample-specific minor variations, yielding a flow of influence from self-awareness (reflective awareness and controlled sense-of-self in the moment) via self-regulation (self-preoccupation and self-compassion) to self-transcendence. Within self-regulation, self-compassion additionally mediated the effects of self-preoccupation in these three data sets. That exact structure was replicated here. It also bears observing that the values of the path coefficients are quite similar across all four studies, suggesting that the S-ART structure is quite stable across samples.

### The structure of the compassion concept

We built our compassion survey out of existing scales for other-directed compassion. This approach allowed us to take a full sweep of the available conceptualizations of the construct. Our factor analysis of the 72 items yielded five interrelated factors, all previously identified in the literature: empathy (as in Gu et al., 2017, based on the theoretical framework by [15], indifference (as in [41], common humanity (as in Gu et al. and Pommier et al.), recognizing suffering (as in Gu et al.), and willingness to act (as in Gu et al.). Missing were the mindfulness and kindness aspects of compassion as defined by Pommier et al., although some of the items on this scale loaded negatively on the indifference factor and one mindfulness item loaded positively on recognizing suffering. Likewise missing was the Gu et al. aspect of tolerating uncomfortable feelings, which was based on the literature review by Strauss et al. Most items of the relevant SOCS-O subscale loaded below the 0.40 threshold on any of the factors, one loaded negatively on indifference.

It seems fair to state that these five aspects map on to Khoury’s [3] trio of affective (empathy and [the reverse of] indifference), cognitive (common humanity, recognizing suffering) and motivational (willingness to act) aspects of compassion. The compassion scales were, as expected, interrelated. It is important to note that indifference is not simply the reverse of the other affective factor, empathy: They do correlate negatively (*r* = − 0.39), but have different antecedents, with indifference being notably more strongly and negatively related to the personality factor of agreeableness and less connected to the mindfulness manifold than empathy is. In fact, the correlation between indifference and agreeableness is so strong (*r* = -0.66) that it could be argued that indifference is not an aspect of compassion at all, but rather simply the opposite of this Big-Five factor.

### From mindfulness over moral foundations to compassion

All in all, compassion was reasonable well explained by the variables included in our set of antecedents, with *R*^2^ ranging from 0.11 to 0.50. At the level of correlations, two aspects of mindfulness correlated consistently with all five aspects of compassion: reflective awareness and self-transcendence. Self-compassion was related to three of the compassion aspects, controlled-sense-of-self in the moment to one, and self-preoccupation to none. Conversely, the aspects of compassion most strongly correlated with mindfulness were empathy, common-humanity, and recognizing suffering.

The path analysis further revealed direct positive links from reflective awareness to the two cognitive aspects of compassion, realizing common humanity and recognizing suffering. Thus, the more active, deliberate, probing aspect of mindfulness is connected to the cognitive aspect of compassion over and beyond its influence through the rest of the S-ART system and the moral foundations. The same added positive influence of reflective awareness was found for empathy, suggesting that at least this affective aspect of compassion is tied to reflection. If we consider indifference as agreeableness in reverse and in disguise rather than a true aspect of compassion, then the conclusion would be that both cognitive and affective aspects of compassion strongly benefit from deliberate self-reflection.

Controlled sense-of-self in the moment was only correlated (and negatively so) with indifference; this relationship was completely mediated through the mindfulness manifold and the moral foundations. In the path analysis, a direct, but negative link was apparent from controlled sense-of-self in the moment to both empathy and common humanity, suggesting that the more passive, equanimous, non-judgmental aspect of mindfulness has less influence on empathy and common humanity than transmitted through S-ART. In sum, this aspect of mindfulness appears to be of lesser importance in understanding compassion.

If we consider mindfulness in its narrow scope of self-awareness, as is traditionally done, we can conclude that the cognitive and affective aspects of compassion are related to the more active, reflective facet of mindfulness, but not to the more passive, observing facet. This finding has implications for intervention: Mindfulness interventions that aim to promote empathy and compassion should probably emphasize the probing aspect of mindfulness rather than invoking the nonjudgmental mind. Some such programs, such as Cognitively-Based Compassion Training [45], which is based on the Tibetan *lojong* tradition, indeed do this explicitly by building in reflective moments geared at instilling a sense of common humanity. Note that this result stands somewhat in contrast to work by Cameron and Fredrickson [46], who found relationships from both observing (akin to reflective awareness) and acceptance (akin to controlled sense-of-self in the moment) to self-reported instances of real-world helping behavior. It is, of course, not clear how such self-reported behavior is ultimately associated with compassion as measured here.

Self-compassion was positively related to empathy and the two cognitive components of compassion. In the path analysis, an additional direct positive path to common humanity was obtained. One simple possibility is that both self-compassion and other-directed compassion rely on similar or overlapping underlying cognitive, affective, or personality characteristics, just with different recipients and different triggers–perceived personal inadequacies for self-compassion, perceived suffering for other-directed compassion [41]. Again, there are implications for intervention here: Interventions that would support self-compassion would then also be likely to foster other-directed compassion.

The finding that self-preoccupation was not significantly correlated with compassion is perhaps not surprising, given that compassion here was explicitly defined as concern for others rather than the self. It is important to note that the close-to-zero correlation (median *r* = 0.07) implies independence rather than a trade-off between self-preoccupation and other-directed compassion. One nuance is noteworthy: In the path model, self-preoccupation presented a negative path to empathy, suggesting that people who are less self-preoccupied also show more empathy than would be expected from the workings of the S-ART model.

The central role of self-transcendence, which was correlated with all compassion aspects, is not surprising, given our previous results with this variable, where we found it to be correlated with almost any beneficial or positive outcome we have ever examined, including eudemonic wellbeing, positive self-view, the moral foundations, the reverse of prejudice, wisdom about self and world, and the three virtues of inquisitiveness, caring, and self-control [10–13]. The path analysis revealed that its influence on compassion goes through the moral foundations,in addition, there is also a direct and positive path to empathy.

Finally, the moral foundations had effects on all aspects of compassion over and beyond these of the mindfulness manifold. Particularly interesting was that individualizing had a beneficial influence on all five compassion measures, whereas binding only influenced the recognition of suffering and the willingness to act (both were positive associations), implying that compassion flows more naturally from the recognition of the importance of fairness and the desire to do no harm than from an ingroup-based moral stance. This appears to be especially true for the affective aspects of compassion.

Finally, we note that the relationship between mindfulness and willingness to act is modest at best, with a median *r* of 0.09, and significant correlations only from reflective awareness and self-transcendence. All the influence was channeled through the binding foundation. Thus, while there are clear links from mindfulness to the affective and cognitive aspects of compassion, the link with the motivational aspect is much less outspoken.

### Awareness of privilege

We included awareness of privilege as an outcome variable because in our prior work, this variable was the only variable whose influence from mindfulness was fully mediated through the moral foundations [13]. We wanted to examine whether this influence in turn would be mediated through compassion. We indeed found this to be the case. The mediation occurred through a cognitive component–common humanity–only, both as an indirect path from the mindfulness manifold and individualizing as well as a direct and positive path. Binding had a direct negative influence on awareness of privilege. This may be a matter of ideology, as binding is often connected to conservative political leanings [13, 47]. Awareness of privilege is a variable of interest at the present moment in history (where it is colloquially known as ‘wokeness’,our findings suggest that both training in mindfulness and in compassion training, as long as it emphasizes common humanity, might be a possible route to enhancing it.

### The role of personality

As in our previous work, we found a clear role for the antecedent variables of personality on the mindfulness manifold and the other variables. In particular, agreeableness had a significant effect on the moral foundations and on all aspects of compassion. This effect was beneficial on all variables, except binding, which was negatively associated with agreeableness, thus indicating that individuals who are highly agreeable put less stock in morality based on group cohesion. Agreeableness has often been shown to be associated with prosocial behavior, including variables examined here, such as empathy (e.g., Melchers et al. [48]), to the point where some consider compassion to be a subtrait of agreeableness [49]. Agreeableness was also positively related to three aspects of the mindfulness manifold, reflective awareness, controlled-sense-of-self in the moment, and self-transcendence. This suggests that some of the shared variance between the mindfulness manifold and compassion would be due to their joint correlation with agreeableness. The path analysis shows that this cannot be the whole story—there are many additional direct and indirect pathways both among the mindfulness variables and between mindfulness and compassion.

### Limitations

The main analysis in this paper was performed using path analysis. Path analysis allows for the examination of a potential flow of influence within a set of variables. An additional advantage of path analysis is that background variables (here: gender and the Big Five) can be included to allow for a clearer view of the relationships between the different constructs after the influence of their potential joint relationship to these background variables has been removed. The cross-sectional nature of the data, however, is an obvious limitation: Longitudinal data, either of an observational nature or gathered from a controlled mindfulness or compassion-focused intervention, would be necessary to fully test the direction of flow.

Additionally, the models are obviously limited by the actual measures used to assess the constructs. The operationalization of the S-ART framework is our own and although this structure has now been replicated numerous times in our own work, we do not how if it extends to different populations and different choices of measures. Likewise, while our measurement of compassion used a large swath of the extant item pool in the literature, a study using more objective metrics would be desirable. Finally, the study is limited by the nature of our sample—US-educated young-adult college students. It remains to be seen if these data patterns generalize to other populations, notably of different age and different cultural background.

## Conclusions

Our factor analysis of the extant surveys on other-oriented compassion revealed distinct affective (empathy, indifference), cognitive (common humanity, recognizing suffering), and motivational (willingness to act) aspects of compassion. Mindfulness, under its aspects of reflective awareness, self-compassion, and self-transcendence, was associated with compassion, with reflective awareness predicting multiple aspects of compassion over and beyond the normal mechanisms of the mindfulness manifold and the moral foundations. The moral foundation of individualizing was associated with all aspects of compassion; the binding foundation was only connected to recognizing suffering and a willingness to act. Awareness of privilege was connected to mindfulness through individualizing and the recognition of common humanity. Thus, mindfulness and compassion have synergistic and distinct effects on ethical sensitivities. Given that both compassion and ethical sensitivities have roots in mindfulness, mindfulness interventions might be a possible venue to enhance these positive aspects of individuals’ psychology.

## Data Availability

The data are publicly available at https://osf.io/vfgz8/.

## Appendix

See Table 3.

**Table 3:** Results of principal component analysis (oblimin rotation), extracting 5 factors from the different compassion questionnaires

Original scale and item number	Item	Empathy	Indifference	Common humanity	Recognizing suffering	Willingness to act
CLH15	If a person (a stranger) is troubled, I usually feel extreme tenderness and caring	0.84				
CLH12	I often have tender feelings toward people (strangers) when they seem to be in need	0.82				
CLH9	I tend to feel compassion for people even though I do not know them	0.82				
CLH6	I feel considerable compassionate love for people from everywhere	0.76				
CLH13	I feel a selfless caring for most of mankind	0.76				
CLH11	I would rather engage in actions that help others, even though they are strangers, than engage in actions that would help me	0.75				
CLH10	One of the activities that provides me with the most meaning to my life is helping others in the world who need help	0.74				
CLH17	I try to put myself in a stranger’s shoes when he or she is in trouble	0.70				
CLH20	I want to spend time with people I don’t know well so that I can help enrich their lives	0.70				
CLH5	If I encounter a stranger who needs help, I would do almost anything I could to help him or her	0.69				
CLH14	I accept others whom I do not know even when they do things I think are wrong	0.67				
CLH3	When I hear about someone (a stranger) going through a difficult time, I feel a great deal of compassion for him or her	0.66				
CLH18	I feel happy when I see that others (strangers) are happy	0.65				
CLH4	It is easy for me to feel the pain (and joy) experienced by others, even though I do not know them	0.63				
CLH2	When I see people I do not know feeling sad, I feel a need to reach out to them	0.61				
CLH8	If given the opportunity, I am willing to sacrifice in order to let the people from other places who are less fortunate achieve their goals	0.59				
CLH7	I would rather suffer myself than see someone else (a stranger) suffer	0.58				
CLH1	When I see people I do not know feeling sad, I feel a need to reach out to them	0.55				
CLH16	I try to understand rather than judge people who are strangers to me	0.53				
CLH19	Those whom I encounter through work and public life can assume that I will be there for them if they need me	0.43				
CS-P3	I am unconcerned with other people’s problems		0.73			
CS-P7	I think little about the concerns of others		0.69			
RCS3	I find it hard to understand other peoples' problems		0.62			
CS-P15	I can’t really connect with other people when they’re suffering		0.61			
CS-P11	I try to avoid people who are experiencing a lot of pain		0.60			
RCS4	I don't know what to do when other people are distressed		0.52			
CS-P1	I pay careful attention when other people talk to me about their troubles		− 0.50			
CS-P9	I listen patiently when people tell me their problems		− 0.50			
CS-P14	When others feel sadness, I try to comfort them		− 0.50			
SOCS-O9	I stay with and listen to other people when they’re upset even if it’s hard to bear		− 0.49			
CS-P2	If I see someone going through a difficult time, I try to be caring toward that person		− 0.48			
RCS1	I like to listen to other peoples' experiences		− 0.43			
SOCS-O8	When I hear about bad things happening to other people, I feel concern for their wellbeing		− 0.43			
SOCS-O4	When someone else is upset, I try to stay open to their feelings rather than avoid them		− 0.42			
CS-P6	I like to be there for others in times of difficulty		− 0.40			
SOCS-O2	I understand that everyone experiences suffering at some point in their lives			0.82		
SOCS-O7	I understand that feeling upset at times is part of human nature			0.82		
SOCS-O12	Like me, I know that other people also experience struggles in life			0.77		
CS-P4	I realize everyone feels down sometimes, it is part of being human			0.70		
SOCS-O17	I know that we can all feel upset at times when we are wronged			0.70		
CS-P16	Despite my differences with others, I know that everyone feels pain just like me			0.60		
CS-P8	I feel it’s important to recognize that all people have weaknesses and no one’s perfect			0.59		
CS-P12	I feel that suffering is just a part of the common human experience			0.58		
CS-P13	When people tell me about their problems, I try to keep a balanced perspective on the situation			0.48		
SOCS-O3	When someone is going through a difficult time, I feel kindly towards them			0.42		
SOCS-O11	I’m quick to notice early signs of distress in others				0.85	
SOCS-O6	I notice when others are feeling distressed				0.84	
SOCS-O1	I recognize when other people are feeling distressed without them having to tell me				0.82	
SOCS-O16	I recognize signs of suffering in others				0.79	
CS-P5	I notice when people are upset, even if they don’t say anything				0.70	
CS-M3	How much of your future savings would you give away now to help a stranger in need of financial help?					0.64
CS-M5	How much of your personal space would you share with a friend?					0.63
CS-M6	How much of your personal space would you share with a stranger that poses no threat to you?					0.60
CS-M4	How much of your free time would you spend to do work for a stranger that needs your skills but cannot afford to pay you?					0.57
CS-M8	How many times would you do the right thing if it puts your family at risk?					0.54
CS-M1	How much of your future savings would you give away now to help a friend in need of financial help?					0.53
CS-M10	How many times would you allow others the pleasure of something that causes you pain?					0.52
CS-M7	How many times would you do the right thing if it puts your friends at risk?					0.49
CS-M2	How much of your future savings would you give away now to help a stranger in need of financial help?					0.48

*n* = 407. CLH = Compassionate Love for Humanity; CS-M = Compassion Scale (Martin); CS-P = Compassion Scale (Pommier); RCS = Relational Compassion Scale; SOCS-O = Sussex-Oxford Compassion for Others Scale. Items not included in the Table had factor loadings below 0.40

## Abbreviations

CLH: Compassionate Love for Humanity
COMP: Compassion
CS-M: Compassion Scale (Martin)
CS-P: Compassion Scale (Pommier)
IPIP: International Personality Item Pool (ipip.ori.org)
MFQ: Moral Foundations Questionnaire
RCS: Relational Compassion Scale
S-ART: Self = awareness, self-regulation, and self-transcendence
S-A: Self-awareness
S-R: Self-regulation
S-T: Self-transcendence
SOCS-O: Sussex-Oxford Compassion for Others Scale
